# 

**Published:** 1879-06

**Authors:** 


					﻿JUNE, 1879.
TWENTY-SIXTH YEAR—N<> 306.
HALL’S
Journal of Health.
PUBLISHED AT 141 EIGHTH STREET,
(Near Broadway),	YORK.
E. H. GIBBS, A.M., M.D., Editor.
AGENTS FOR THE TRADE I
AMERICAN NEWS COMPANY AND NEW YORK NEWS COMPANY.
Terms, $1.50 a Tear, or $1.00 for Eight Months. Postage paid
SINGLE NUMBER, IB CENTS.
Couch & Johnston, Printers, 11 Frankfort Street. New York.
				

